# PKR knockout in the 5xFAD model of Alzheimer's disease reveals beneficial effects on spatial memory and brain lesions

**DOI:** 10.1111/acel.12887

**Published:** 2019-03-01

**Authors:** Marion Tible, François Mouton Liger, Julien Schmitt, Albert Giralt, Karim Farid, Sylvie Thomasseau, Sarah Gourmaud, Claire Paquet, Laure Rondi Reig, Eliane Meurs, Jean‐Antoine Girault, Jacques Hugon

**Affiliations:** ^1^ Inserm U1144 Paris France; ^2^ Inserm U1127 Paris France; ^3^ Institut de Biologie Paris Seine CNRS, UMR 8246 Paris France; ^4^ Inserm U1130 Paris France; ^5^ Sorbonne Université Paris France; ^6^ Inserm U839 Paris France; ^7^ Institut du Fer à Moulin Paris France; ^8^ Department of Nuclear Medicine CHU Fort de France Martinique France; ^9^ Center of Cognitive Neurology, Lariboisière Fernand Widal Hospital APHP Paris France; ^10^ Perelman School of Medicine University of Pennsylvania Philadelphia Pennsylvania; ^11^ Paris Diderot University Paris France; ^12^ Hepacivirus and Innate Immunity Unit Institut Pasteur Paris France; ^13^ CNRS, UMR 3569 Paris France

**Keywords:** Alzheimer, amyloid, memory, neurodegeneration, neuroinflammation, PKR

## Abstract

Brain lesions in Alzheimer's disease (AD) include amyloid plaques made of Aβ peptides and neurofibrillary tangles composed of hyperphosphorylated tau protein with synaptic and neuronal loss and neuroinflammation. Aβ oligomers can trigger tau phosphorylation and neuronal alterations through activation of neuronal kinases leading to progressive cognitive decline. PKR is a ubiquitous pro‐apoptotic serine/threonine kinase, and levels of activated PKR are increased in AD brains and AD CSF. In addition, PKR regulates negatively memory formation in mice. To assess the role of PKR in an AD in vivo model, we crossed 5xFAD transgenic mice with PKR knockout (PKRKO) mice and we explored the contribution of PKR on cognition and brain lesions in the 5xFAD mouse model of AD as well as in neuron–microglia co‐cultures exposed to the innate immunity activator lipopolysaccharide (LPS). Nine‐month‐old double‐mutant mice revealed significantly improved memory consolidation with the new object location test, starmaze test, and elevated plus maze test as compared to 5xFAD mice. Brain amyloid accumulation and BACE1 levels were statistically decreased in double‐mutant mice. Apoptosis, neurodegeneration markers, and synaptic alterations were significantly reduced in double‐mutant mice as well as neuroinflammation markers such as microglial load and brain cytokine levels. Using cocultures, we found that PKR in neurons was essential for LPS microglia‐induced neuronal death. Our results demonstrate the clear involvement of PKR in abnormal spatial memory and brain lesions in the 5xFAD model and underline its interest as a target for neuroprotection in AD.

## 1 INTRODUCTION

In Alzheimer's disease (AD), amyloid β (Aβ) oligomers are hypothesized to trigger tau phosphorylation and neuronal alterations by activating kinases (Duyckaerts, Delatour, & Potier, 2009; Selkoe & Hardy, 2016), leading to progressive cognitive decline (Scheltens et al., 2016). One such kinase is protein kinase R (PKR), a ubiquitous pro‐apoptotic serine/threonine kinase that participates in the “integrated stress response” (ISR)(Marchal et al., 2014) (Patel & Sen, 1998) (Shimazawa, Ito, Inokuchi, & Hara, 2007) (Harding et al., 2003), a cell stress response key to cell homeostasis. PKR is activated in AD brains (Chang, Suen, et al., 2002; Chang, Wong, Ng, & Hugon, 2002; Hugon, Mouton‐Liger, Dumurgier, & Paquet, 2017; Page et al., 2006; Paquet et al., 2012), and its concentration in the cerebrospinal fluid (CSF) predicts cognitive decline (Dumurgier et al., 2013; Mouton‐Liger, Paquet, Dumurgier, Lapalus, et al., 2012). PKR activation can control tau phosphorylation (Bose et al., 2011; Suen, Yu, So, Chang, & Hugon, 2003), the expression of the Aβ‐forming protein BACE1 (Ill‐Raga et al., 2011; Mouton‐Liger, Paquet, Dumurgier, Bouras, et al., 2012), and neuroinflammation linked to innate immunity (Kang & Tang, 2012). More information can be found in a recent review on PKR and AD (Hugon et al., 2017). PKR impairs memory formation in mice (Zhu et al., 2011). No study has explored the genetic blockade of PKR in AD transgenic mice. Here, we investigated the role of PKR in the cognitive alterations that accompanies AD lesions by crossing the 5xFAD mouse model of AD, mice that express five human familial AD mutations (Oakley et al., 2006), and PKR knockout mice (PKRKO) (Yang et al., 1995). We assessed for the first time, spatial memory, brain amyloid, apoptosis, and neuroinflammation in WT, 5xFAD, PKRKO, and double‐mutant mice. The double‐mutant mice displayed improvement in spatial memory, decreased brain amyloid accumulation and BACE1 activity, and reduced apoptosis and inflammation as compared to 5xFAD mice. In addition, using neurons and microglial cell cocultures, we found that PKR in neurons was key for microglia‐induced neuronal death. Our results highlight the potential for this kinase as a target for neuroprotection.

## 2 RESULTS

### 2.1 PKR genetic blockade reduces memory impairment in 5xFAD mice

**Figure 1:**
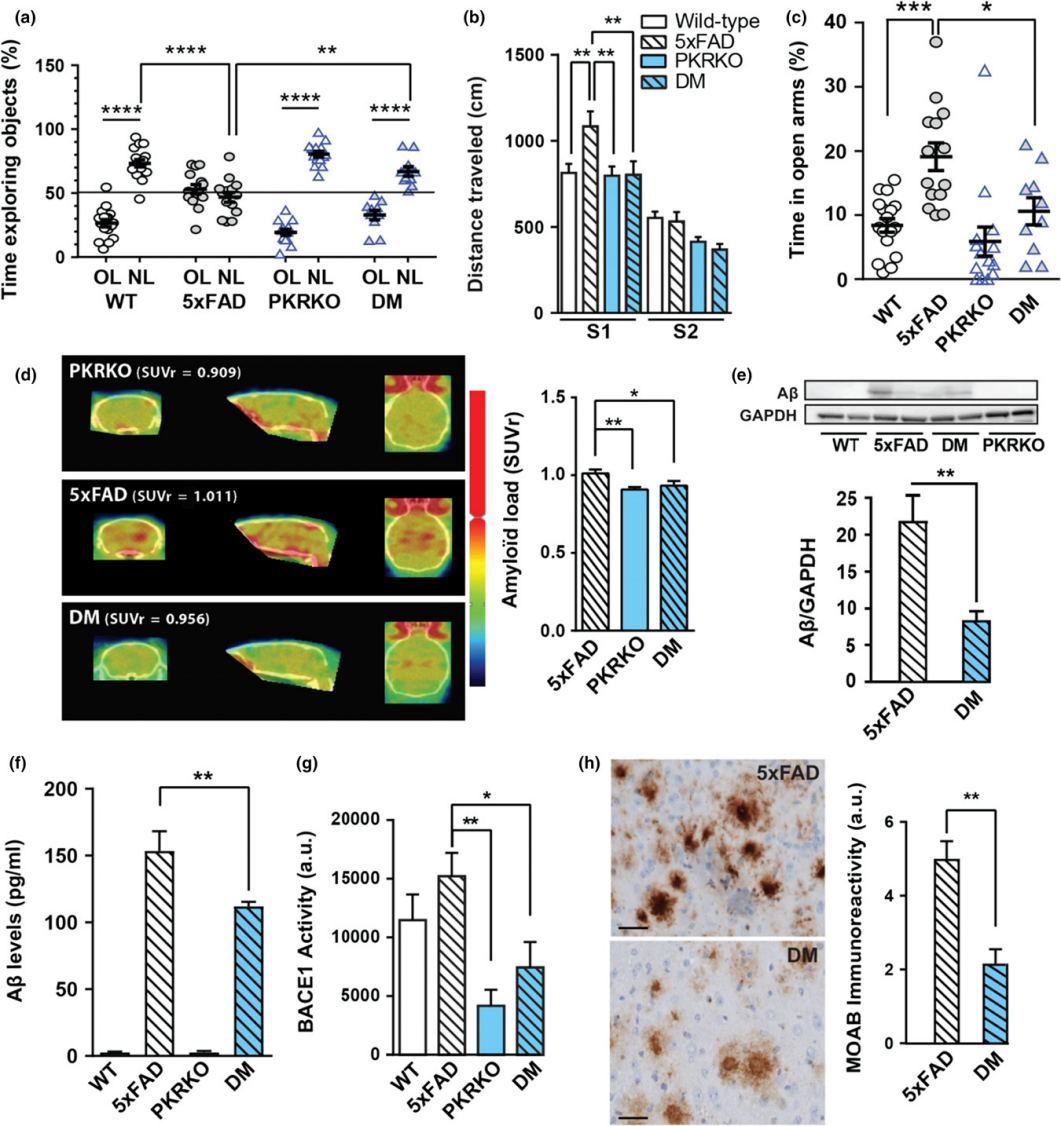
PKR knockout modulates cognition and Aβ formation. (a–c) Behavior was studied in 9‐month‐old male and female wild‐type (*n* = 17), 5xFAD (*n* = 14), PKRKO (*n* = 14), and 5xFAD × PKRKO (double mutant, DM, *n* = 10) mice. (a) In the novel object location test, the percentages of time exploring the displaced object (new location, NL, 24 hr after first exposure) and the unmoved object (old location, OL) were compared. Two‐way ANOVA interaction *F*_(3,51)_ = 21.06, *p* < 0.0001, OL vs. NL, Holm–Sidak's test, *p* < 0.0001 for wild‐type, nonsignificant for 5xFAD, *p* < 0.0001 for PKRKO, and *p* < 0.0001 for DM. (b) Performances in the starmaze. S1, S2 = session number (5 trials per session). Repeated measures ANOVA, genotype effect *p* = 0.0184, sessions effect *p* < 0.0001, interaction *F*_(7,100)_ = 18.96, post hoc Holm–Sidak's test. (c) Anxiety assessment in the elevated plus maze. Time in open arm between 5xFAD and double‐mutant mice: Kruskal–Wallis test *p* < 0.0001, post hoc Mann–Whitney test. (d) PET scan analysis of amyloid burden in PKRKO, 5xFAD, and DM mice (*n* = 4–6 per group). All results are expressed as the standard uptake value ratio (SUVr) relative to the cerebellum. (e, f) Quantification of Aβ levels in the hippocampus of 30‐week‐old mice by immunoblot analysis (e) with GAPDH used as the loading control, and by ELISA analysis (f) (*n* = 6 mice per group). Kruskal–Wallis, *p* < 0.0001, Dunn's post hoc test. (g) BACE1 levels in the hippocampus of 30‐week‐old mice (*n* = 10 mice per group). Kruskal–Wallis, *p* = 0.0011, Dunn's post hoc test. (I) Aβ immunohistochemistry with Moab antibody, revealed with horseradish peroxidase, in the subiculum of 30‐week‐old mice (*n* = 5 mice per group). Significant reduction of Aβ load in double‐mutant mice after quantification. Mann–Whitney test, *p* = 0.016. (a–h) Data are means ± *SEM*. *p < 0.05, **p < 0.01, ***p < 0.001, ****p < 0.0001

We used 5xFAD transgenic mice (9‐month‐old) that show amyloid plaques deposition starting at 2 months and widespread neuronal degeneration maximal in the subiculum and layer 5 of the cortex at 9 months (Oakley et al., 2006). PKR was absent in the brains of PKRKO mice and double‐mutant mice (Supporting information Figure S1). First, we examined the spatial memory of the mice using the novel object location test (Ennaceur, Neave, & Aggleton, 1997), in which 24 hr after mice are familiarized with two identical objects; one of the objects is placed at a different location. Wild‐type and PKRKO mice spent more time exploring the displaced object than the unmoved object (Figure 1a). As expected, this preferential exploratory behavior was absent in 5xFAD mice, which could not distinguish between the displaced versus the unmoved objects (Figure 1a). In contrast, the double‐mutant mice spent more time exploring the displaced object, showing a statistically significant improvement (44%) in spatial memory after KO of PKR as compared to 5xFAD mice. In the starmaze exploration (Rondi‐Reig et al., 2006), a cognitive test that distinguishes different memories and assessed by the length of travel to the target, the distance travelled by 5xFAD mice in the first session was increased as compared to double‐mutant mice (Figure 1b). Only PKRKO mice had significantly reduced numbers of errors compared to the other three groups (Supporting information Figure S2). As a normal level of anxiety is crucial for the behavioral control required during spatial learning, we also evaluated anxiety responses in the four groups of mice using the elevated plus maze. The time spent in the open arm of the elevated plus maze was increased in the 5xFAD mice (Figure 1c), suggesting that these animals display impaired anxiety responses, in agreement with previously published results (Jawhar, Trawicka, Jenneckens, Bayer, & Wirths, 2012). This decrease in anxiety returned to levels observed in wild‐type animals in the double‐mutant mice (Figure 1c). These results suggest that PKR deletion partially rescues the spatial memory deficit observed in the 5xFAD model of AD.

### 2.2 Reduction of brain amyloidosis by PKR blockade

Since amyloid accumulation is toxic to neurons and PKR can control BACE1 expression, we next evaluated the effects of PKR knockout on brain amyloid deposition. To this end, we first used the amyloid marker ^18^F‐florbetaben (Neuraceq®) for positron‐emission tomography (PET) in live 5xFAD, PKRKO, and double‐mutant mice. The mean standard uptake value ratio (SUVr) of the 5xFAD mice was statistically higher than the SUVr of the double‐mutant and PKRKO mice (Figure 1d). Brain amyloid load and SUVr were reduced in PKRKO mice and not statistically different from double‐mutant mice. Reduction of Aβ levels in the double‐mutant mice compared to the 5xFAD mice was confirmed using immunohistochemistry (Figure 1e), Aβ immunoblotting (Figure 1f), and ELISA method (Figure 1g). In addition, BACE1 levels were significantly reduced (−176%) in the brains of double‐mutant mice as compared to 5xFAD mice. These levels were also significantly reduced in PKRKO mice as compared to WT mice (Figure 1h). These various measurements show that the absence of PKR results in a reduced amyloid accumulation and BACE1 levels in 5xFAD mice. The observed reduction in brain BACE1 concentrations in double‐mutant mice may be a direct result of absence of PKR activity, which has been shown to regulate BACE1 expression at the post‐transcriptional level (Ill‐Raga et al., 2011; Mouton‐Liger, Paquet, Dumurgier, Bouras, et al., 2012), and/or could result from a diminished activity of PKR‐induced kinases, such as C‐Jun‐N‐terminal kinase (JNK) or P38 MAP kinase, which regulate BACE1 expression at the transcriptional level (Tamagno et al., 2005). This will deserve further investigation.

### 2.3 Decrease in neurodegeneration induced by PKR genetic blockade

**Figure 2:**
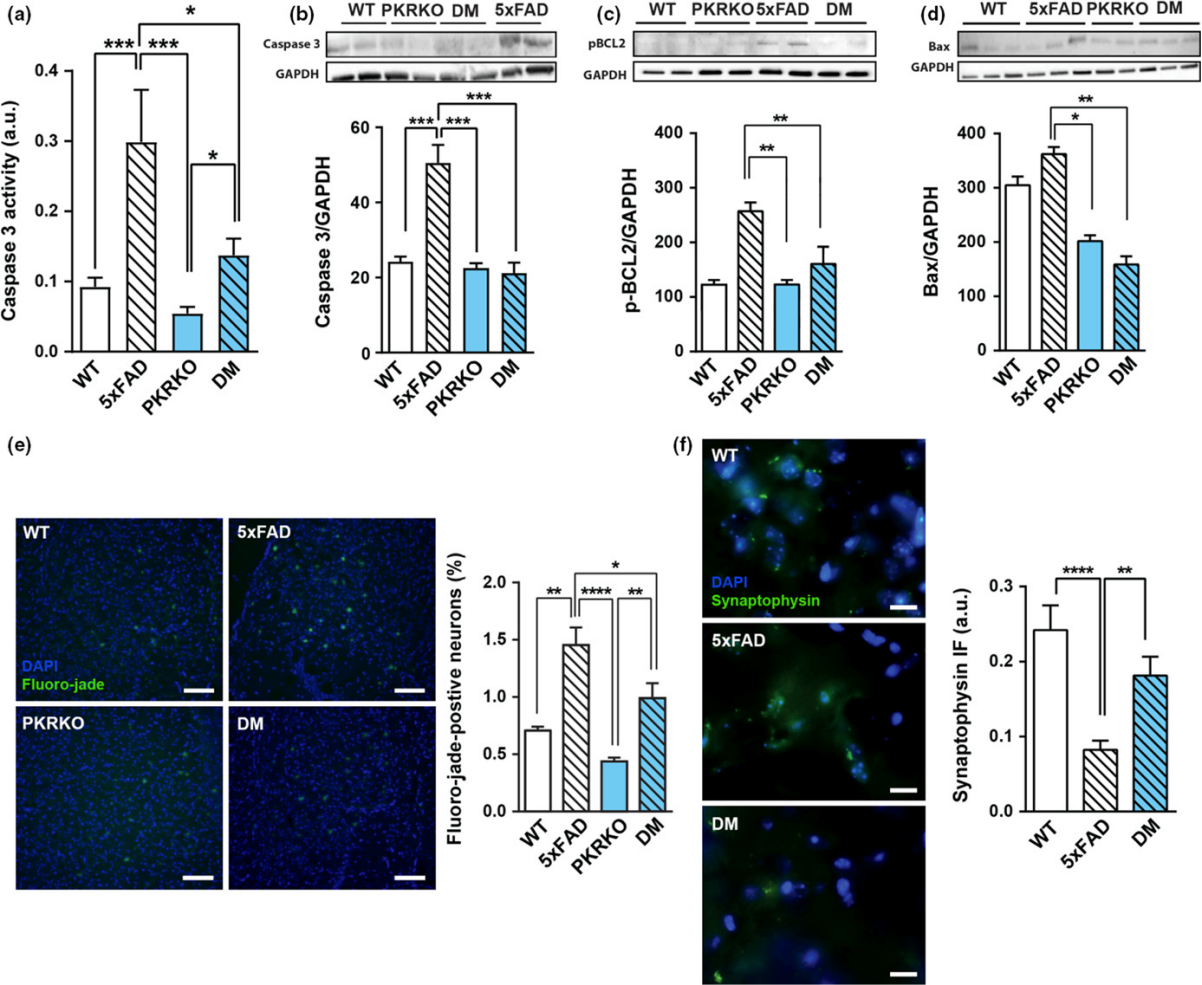
PKR knockout enhances neuroprotection in 5xFAD mice. (a) Caspase 3 activity in the hippocampus of 30‐week‐old wild‐type, 5xFAD, PKRKO, and 5xFAD×PKRKO (double mutant, DM) mice (*n* = 10 mice per group). Two‐way ANOVA, *F*_(3,36)_ = 3.718, *p* = 0.0025; Tukey's post hoc test. (b–d). Immunoblotting analysis of caspase 3 (b), phospho‐bcl2 (c), and BAX (d) in the hippocampus of 30‐week‐old mice (*n* = 6 mice per group). GAPDH was used as the loading control. (b) One‐way ANOVA, *F*_(3,16)_ = 1.546, Tukey's post hoc test, *p* < 0.0001. (c) Kruskal–Wallis, Dunn's post hoc tests, *p* = 0.001. (d), One‐way ANOVA, *F*_(3,29)_ = 0.2476, *p* = 0.0014, Tukey's post hoc test. (e) Fluoro‐jade C fluorescence in the subiculum of 30‐week‐old mice (*n* = 6 mice per group) (horizontal bar 50 µM). One‐way ANOVA, *F*_(3,19)_ = 3.811, *p* = 0.0001, Tukey's post hoc test. (f) Synaptophysin immunofluorescence in the subiculum of 30‐week‐old wild‐type, 5xFAD, and DM mice (*n* = 5 mice per group). (horizontal bar 20 µM) Kruskal–Wallis test, *p* < 0.0001, Dunn's post hoc test. (a–f) Data are means ± *SEM* *p < 0.05, **p < 0.01, ***p < 0.001, ****p < 0.0001

Memory deficits in 5xFAD mice can be linked to synaptic and neuronal degeneration in the hippocampus and in the cortex (Oakley et al., 2006) as also observed in human AD brains. We biochemically evaluated brain apoptotic markers (caspase 3, phospho‐bcl2, Bax), neuronal degeneration (Fluoro‐jade staining), and synaptic alterations (synaptophysin and PSD‐95) in the four groups of mice. Caspase 3 activity (Figure 2a) and levels (Figure 2b) were significantly reduced in the hippocampus of double‐mutant mice as compared to 5xFAD mice. Phospho‐bcl2 and BAX levels (Figure 2c–d) were also significantly decreased in the hippocampus of double‐mutant mice compared to 5xFAD mice, revealing that PKR is essential for the activation of these three apoptotic markers leading to neuronal degeneration as previously shown (Shimazawa et al., 2007). A trend toward increased caspase 9 levels was observed in 5xFAD, but not in double‐mutant mice as compared to WT mice (Supporting information Figure S3). These results reveal reduced neuronal apoptosis in the absence of PKR in 5xFAD mice; therefore, we next evaluated neurodegeneration in the four group of mice with Fluoro‐jade staining. Histological quantifications confirmed apoptosis results as it revealed significantly reduced levels of staining in the subiculum of double mutants compared to 5xFAD mice (Figure 2e). In addition, synapse number, as measured by immunofluorescence labeling for presynaptic protein synaptophysin (Figure 2f) and postsynaptic PSD‐95 (Supporting information Figure S4), was also significantly decreased in 5xFAD mice, compared to WT and double‐mutant mice. Collectively, these results indicate that the absence of the brain pro‐apoptotic PKR results in reduced apoptosis and reduced synaptic and neuronal degenerations in 5xFAD mice.

### 2.4 Reduction of neuroinflammation induced by PKR blockade

**Figure 3:**
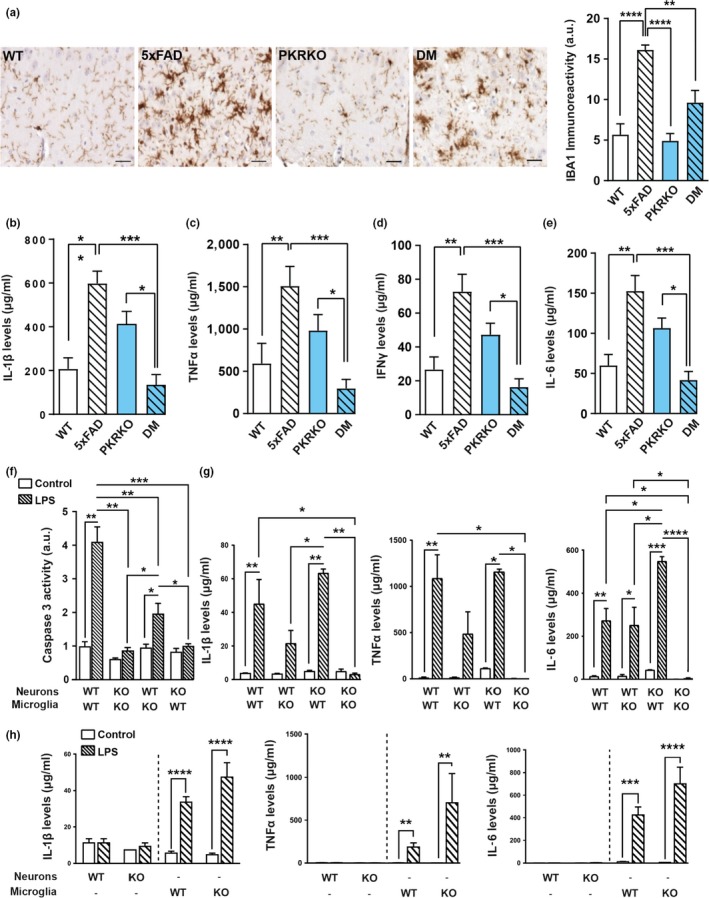
PKR knockout reduces neuroinflammation in 5xFAD mice. (a) IBA1 immunohistochemistry in the subiculum of 30‐week‐old wild‐type, 5xFAD, PKRKO, and 5xFAD×PKRKO (double mutant, DM) mice (*n* = 5 mice per group) (horizontal bar 20 µM). Quantitative results show a significant reduction of IAB1 immunostaining in double‐mutant mice as compared to 5xFAD mice. One‐way ANOVA, *F*_(3,16)_ = 0.5339, *p* < 0.0001, Tukey's post hoc test. (b–e) Luminex analysis of IL‐1β (b), TNFα (c), IFNγ (d), and IL‐6 (e) levels in the hippocampus of 30‐week‐old wild‐type, 5xFAD, PKRKO, and 5xFAD×PKRKO (double mutant, DM) mice (*n* = 6 mice per group). Kruskal–Wallis test, (c) *p* = 0.0001, (d–e), *p* < 0.0001, Dunn's post hoc test. (f–h) Neurons and microglia derived from wild‐type or PKRKO mouse embryos, as indicated, were cocultured and treated for 24 hr with 1 µg/ml LPS. Caspase 3 activity was measured in the cell lysates at the end of the treatment (*n* = 3–5 cocultures per group). One‐way ANOVA, *F* (7, 22) = 2.936, *p* < 0.0001; Tukey's post hoc test. Neurons and microglia from wild‐type or PKRKO embryos were cocultured as in f, or cultured alone as indicated. Cytokine levels were measured using Luminex in the supernatant 1 day after a 24‐hr treatment with vehicle (control) or 1 µg/ml LPS. IL‐1β, TNFα, and IL‐6 (g: cocultures)(h; pure cultures). Two‐way ANOVA, interaction *F* (3, 27) = 3.935, *p* = 0.0189; treatment effect *F* (3, 27) = 4.010, *p* = 0.0175; and genotype effect *F* (1, 27) = 22,47, *p* < 0.0001, respectively; Tukey's post hoc test. (a–i) Data are means ± *SEM*, *p < 0.05, **p < 0.01, ***p < 0.001, ****p < 0.0001

The role of PKR in neurodegeneration could also be linked to inflammation as observed in human AD brains (Carret‐Rebillat et al., 2015; Dabo & Meurs, 2012). We therefore explored the consequences of PKR deletion on various brain inflammatory markers in the four groups of mice. Quantification of IBA1, a microglial protein, by immunohistochemistry revealed a significant reduction in staining in the subiculum of double‐mutant mice compared to 5xFAD mice (Figure 3a). Using the Luminex method, we next measured the levels of several proinflammatory cytokines, namely IL‐1β, TNFα, IFNγ, and IL‐6, in the hippocampus of the four groups of mice and found that they were all significantly increased in 5xFAD mice returning to wild‐type levels in double‐mutant mice (Figure 3b–e). Surprisingly, in the brains of PKRKO mice a trend to increased cytokine levels was detected. This finding could be linked to an enhanced regulation of PKR‐independent inflammatory pathways in the absence of PKR (Kang & Tang, 2012). All these results suggest a role for PKR in neuroinflammation in 5xFAD mice. Indeed, reduced inflammation has been observed in PKRKO mice following LPS injection as compared to wild‐type mice (Carret‐Rebillat et al., 2015). Moreover, pharmacological PKR inhibition can reduce IL‐1β levels in a rat model of excitotoxicity and neuroinflammation (Tronel, Page, Bodard, Chalon, & Antier, 2014), but our results show for the first time that PKR genetic blockade reduces neuroinflammation in an AD transgenic mice model.

### 2.5 PKR activation modulates inflammation and neuronal death in cultures

Next, we examined whether the observed reduction in neuroinflammation and apoptosis in the absence of PKR was due to neuronal or microglial PKR. As a model, we used in vitro LPS‐induced microglial cell activation. To this end, release of proinflammatory cytokines and apoptosis were measured upon LPS treatment (1 µg/ml for 24 hr) of cultured neuronal and microglial cells isolated from wild‐type and PKRKO mice. No cytokine release was observed in neuronal cultures from either mouse strain, as expected, whereas robust release was detected in both wild‐type and PKRKO microglial cultures treated with LPS (Figure 3h and Supporting information Figure S5). The result confirms that neurons do not respond to LPS exposure. The LPS‐induced cytokine release from PKRKO‐derived microglia suggests that inflammation in this model was triggered through PKR‐independent pathways (Dabo & Meurs, 2012). To determine whether neuronal and/or microglial PKR was involved in inflammation‐induced neuronal death, we next prepared mixed neuronal and microglial cocultures from either wild‐type or PKRKO mice to test the neurotoxicity of LPS‐triggered microglial activation. LPS treatment resulted in a fourfold increase in caspase 3 activity in wild‐type cocultures, whereas it resulted in just a twofold increase in cocultures of PKRKO‐derived microglia and wild‐type neurons, and was completely abolished in cocultures containing PKRKO‐derived neurons (Figure 3f). These results indicate that although PKR in both neurons and microglia is involved in caspase 3 activation, only neuronal PKR plays an essential role in neuronal apoptosis. Neurons devoid of PKR are protected against microglial activation. To further assess microglial activation, we measured cytokine release in the supernatant of the cocultures. Only cocultures containing both PKRKO‐derived neurons and microglia demonstrated a complete blockade of LPS‐induced release of IL‐1β, IL‐6, TNFα, IFNγ, and IL‐10 (Figure 3g and Supporting information Figure S5).

As mentioned in the introduction, PKR can induce ISR and ISR proteins are triggered after activation of all eIF2 kinases. Surprisingly, no statistical difference was observed for other proteins of the ISR including phosphorylated eIF2α, GADD34, CHOP1, and ATF4 levels between 5xFAD mice and double‐mutant mice (Supporting information Figure S6). This finding has also been very recently observed in another report (Sadleir, Popovic, & Vassar, 2018). These results suggest that ISR could occur earlier in 5xFAD transgenic mice or that ISR‐independent pathways activated by PKR such as NF‐κB activation (Bonnet, Weil, Dam, Hovanessian, & Meurs, 2000) could also play a major role.

## 3 DISCUSSION

Our results demonstrate for the first time that the deletion of the PKR gene in a transgenic model of AD is able to reduce several functional and pathological brain alterations observed in the 5xFAD model. Indeed, PKR knockout improved cognitive abilities in 9‐month‐old AD mice and led to improvements in biochemical and histological markers of AD. It is striking to notice that functionally, biochemically, and neuropathologically, the absence of PKR attenuates most of the abnormal features observed in the severe AD mice model 5xFAD (Oakley et al., 2006). So far, no PKR inhibitor has been tested in human clinical trials but the possibility to interfere broadly with several AD brain lesions by PKR alterations could lead in the future to a pharmacological development of new and efficient PKR modulators. Abnormal PKR levels seem to be already present in the brains and CSF of MCI due to AD patients (Mouton‐Liger, Paquet, Dumurgier, Lapalus, et al., 2012). This finding leads to the conclusion that early MCI should be the more appropriate phase to carry out PKR inhibition in affected patients with AD brain lesions.

Several conclusions can be drawn from our findings:

The absence of PKR has a positive effect on memory in experimental animals (Abraham & Williams, 2008; Jiang et al., 2010), and our results demonstrate for the first time these findings in a severe AD transgenic model. It is not possible so far to determine whether the PKR genetic blockade interferes with memory processes or whether the memory improvement is due to reduced brain lesions in double‐mutant mice or whether both mechanisms are involved. It is not impossible to postulate that PKR inhibition could have a symptomatic effect as well as a disease‐modifying action in 5xFAD mice. One will have to wait for future human clinical trials to determine whether this dual action could be present.

**Figure 4:**
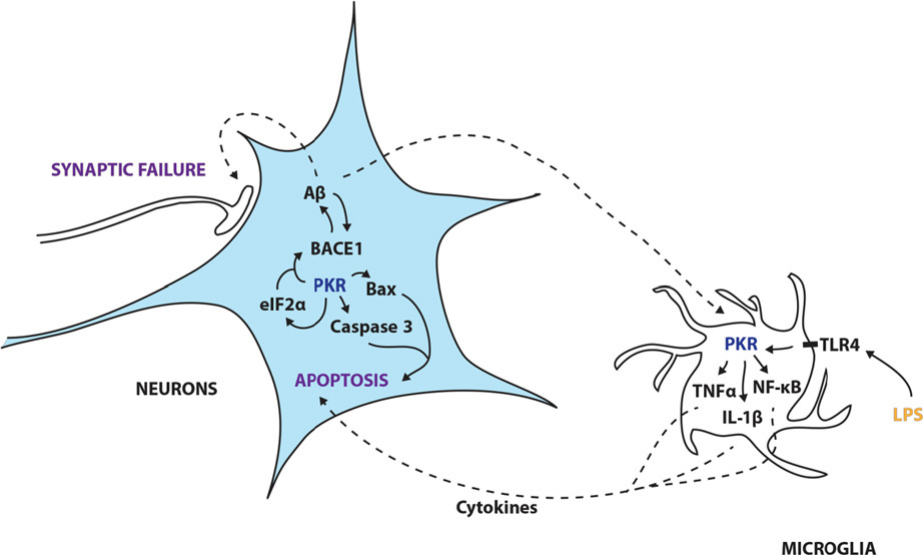
Signaling pathways in Alzheimer's disease in which PKR is implicated. Schematic representation of the pathways PKR may modulate based on the current and previous findings in neurons and microglia. In microglia, Aβ or LPS can trigger a PKR‐dependent production of cytokines (such as TNFα or IL‐1β) that are secondarily released or a PKR‐dependent activation of NF‐κB leading to inflammation. In neurons, cytokines from microglia or Aβ oligomers can trigger a PKR‐dependent increase in BACE1 activity, ISR activation via eIF2α, and a PKR‐dependent apoptosis via phospho‐bcl2 and BAX

We showed in this report that blocking PKR activity could modulate simultaneously spatial memory, amyloidosis, apoptosis, synaptic loss, and neuroinflammation (Figure 4). Early reduction of PKR activity could have a multitarget impact in human affected by AD brain lesions especially if this action could be undertaken in early MCI patients. One can propose that, in addition to follow the patients treated with PKR inhibitors with cognitive evaluation, other surrogate makers could be used such as brain amyloid load and CSF neurodegenerative biomarkers.

Aβ oligomers can induce cytotoxic microglial activation (Selkoe & Hardy, 2016), and we show in vitro for the first time that neuronal PKR activation is crucial as compared to microglial PKR in neuronal apoptosis triggered by cytokine release. Nevertheless, microglial and neuronal PKR are both valid targets to pharmacologically reduce neuroinflammation, synaptic loss, and neuronal apoptosis. This finding argues in favor of a general PKR inhibition if an appropriate inhibitor is found. ISR triggering has not been biochemically detected in 9‐month‐old 5xFAD mice (Harding et al., 2003). Further work will be needed to explore early stages in double‐mutant mice to decipher the role of purely PKR‐dependent and/or also ISR‐dependent neuronal alterations, neuroinflammation, and amyloidosis. The presence of ISR and unfolded protein response in postmortem human brains (Scheper & Hoozemans, 2015) does not indicate evidently when this process has started in AD patients but is an indication that targeting PKR as early as possible might contribute to attenuate many detrimental consequences of PKR activation in affected individuals. A recent report has shown that targeting the other ISR kinase PERK can modulate also memory and attenuate neurodegeneration in other experimental models (Mercado & Hetz, 2017). Similarly, new PKR inhibitors are now experimentally tested and have shown neuroprotective properties and can reduce brain amyloid accumulation (Mouton‐Liger et al., 2015).

Our study has some limitations. Neurofibrillary tangles are absent in the 5xFAD model, and a first limitation of this study is that the modulation of tau phosphorylation by blocking PKR could not be assessed. Although previous experimental works have demonstrated that PKR modulates tau phosphorylation (Bose et al., 2011), further in vivo works with appropriate tau transgenic models will be necessary. The 5xFAD Alzheimer mice is a rapidly evolving transgenic model, and the abnormal brain molecular pathways in these mice could be somewhat different from the more slowly progressing lesions observed in sporadic AD. These mice have also a possible genetic heterogeneity, and the use of another AD transgenic mice could be worth trying. Finally, as soon as a nontoxic PKR inhibitor will be available for long‐term treatments, a pharmacological confirmation of our findings will be necessary.

In conclusion, PKR knockout has beneficial effects in a mouse model of severe AD; this likely reflects its involvement at multiple levels in the pathogenesis of the disease. Because the absence of PKR is well tolerated in PKRKO mice (Yang et al., 1995) without overt histopathological lesions, modulating PKR may be a promising approach to improve cognition and attenuate the brain lesions observed in AD.

## 4 EXPERIMENTAL PROCEDURES

### 4.1 Mice

All mice were bred and housed in controlled environmental conditions with free access to water and food at the TAAM—CNRS UPS 44—CDTA (Orléans, France). Wild‐type (littermates of the 5XFAD strain), 5xFAD, PKRKO, and 5xFAD/PKRKO mice were all of a C57BL/6 background and 9 months of age at the time of the analyses. 5xFAD heterozygous mice overexpress transgenic human amyloid precursor protein (APP) with the Swedish, Florida, and London mutations and PSEN1 with M146L and L286V mutations, both driven by the mouse Thy1 promoter (Oakley et al., 2006). PKRKO homozygous mice do not express PKR following deletion of coding exons 2 and 3 (Mouton‐Liger, Paquet, Dumurgier, Lapalus, et al., 2012). 5xFAD/PKRKO mice were obtained by crossing the two lines. All mice were genotyped for the presence of FAD mutations and/or the absence of the PKR gene. Experimental protocols were approved by the regional ethical committee of the University of Paris 7, France.

### 4.2 Behavioral tests

The novel object location (NOL) test was performed as previously described (Ennaceur et al., 1997). Briefly, mice were first habituated to the open‐top arena (45 × 45 × 45 cm, 2 days, 15 min per day). On day 3, two identical objects (A1 and A2) were placed in the arena and explored for 10 min. Twenty‐four hours later, one object was moved from its original location to the diagonally opposite corner, and mice were allowed to explore the arena for 5 min. The object preference was measured as the time exploring each object ×100/the time exploring both objects.

The starmaze consisted of five alleys radiating from the vertices of a central pentagonal ring. All the alleys were filled with water, and the water was made opaque with an inert nontoxic product (AccuScan OP 301, Brenntag, Lyon, France). The maze was surrounded by a square black curtain with 2D and 3D patterns affixed to provide configurations of spatial cues. To avoid the possible use of a guidance strategy (i.e., animals could rely on the use of a single distal cue), cue was given in duplicate. White noise was used to cover all other sounds that the mice could have used to orientate themselves. To solve the task, animals had to swim to a platform hidden 1 cm below the water surface and located 10 cm from the end of one alley. Departure and arrival points were always the same. All animals ran one session of five trials per day over 2 days using a 40‐min intertrial interval. If an animal did not locate the escape platform within 90 s, the experimenter placed the animal onto the platform, where it remained for 30 s. During the protocol, one central alley and two peripheral alleys were blocked, forcing the mice to use the “left pathway.” Mice were tracked by using the Smart Software (Bioseb, Vitrolles, France).

The elevated plus maze consisted of two opposing 30 × 8 cm open arms and two opposing 30 × 8 cm arms enclosed by 15‐cm‐high walls. The maze was raised 50 cm above the floor and lit with dim light. Each mouse was placed in the central square, facing an open arm, and the time spent in the open arms was recorded for 5 min.

### 4.3 Brain tissue preparation

Briefly, mice were deeply anesthetized with a lethal dose of pentobarbital, and a cannula was inserted in the heart. Mice were perfused with cold PBS. The hippocampus and cortex from one hemibrain section were frozen. The other hemibrain sections were immersed in 4% paraformaldehyde (Sigma‐Aldrich, MO, USA) followed by inclusion of samples in paraffin.

### 4.4 Cell cultures

Cell culture medium was prepared as previously described (Page et al., 2006) and consisted of DMEM with 10% FBS (for microglia) and neurobasal supplemented with GlutaMAX, 50 μg/ml penicillin, 10 ng/ml streptomycin, and B27 (R&D Systems, USA) (for neurons). Mouse embryonic neurons and microglia were extracted from (14 days) embryo cortexes collected from pregnant 5xFAD or wild‐type female mice and were plated onto poly‐L‐ornithine‐coated (microglia) or poly‐L‐lysine‐coated (neurons) plates (Sigma‐Aldrich, Germany) with the above‐described culture medium. Cells were used at 14 days (microglia) or 7 days (neurons) after seeding. For cocultures, 7‐day‐old in vitro neurons were transferred to plates containing 14‐day‐old in vitro microglial cells. LPS (1 µg/ml) (Sigma‐Aldrich, Germany) was applied for 24 hr, followed by cell culture washings. The following day, cells were used either for immunostaining or, after extraction with trypsin‐EDTA (Life Technologies, USA), for immunoblotting. The supernatant was always collected before either type of analysis and used for enzymatic assays and Luminex analyses.

### 4.5 Immunostaining of cultured cells

Cells were fixed with 40 g/L formaldehyde (Solveco, Sweden) for 15 min, permeabilized with 5 µl/ml Triton X‐100 (Sigma‐Aldrich, Germany) for 10 min, and blocked with 50 g/L BSA (Life Technologies, USA) and 1 µl/ml Tween‐20 (Sigma‐Aldrich, Germany) for 1 hr. The protocol for TUNEL analysis was then carried out according to the manufacturer's guidelines (ApopTag® Peroxidase In Situ Apoptosis Detection Kit, EMD Millipore).

### 4.6 Immunoblotting

Antibodies for Aβ (Mouse monoclonal 3G5; Thermo Fisher, Waltham MA, USA), total PKR (rabbit polyclonal antibody 18,244–1‐AP 1/1,000; Proteintech, Rosemont, IL, USA), cleaved caspase 3 (rabbit monoclonal, 5A1E, Cell Signaling), phospho‐bcl2 (mouse monoclonal antibody C‐2, Santa Cruz Biotechnology) and BAX (rabbit monoclonal antibody D2E11, Cell Signaling) were used to carry out western blotting according to the suppliers' instructions. Briefly, proteins were extracted either from a cell culture or a mouse hippocampus using RIPA (RadioImmunoPrecipitation Assay) complemented with a 1/20 dilution of a cocktail of protease inhibitors (SIGMAFAST™, S8820, Sigma‐Aldrich), a 1/100 dilution of calyculin and a 1/1,000 dilution of orthovanadate, and the protein concentration was measured using BCA (BiCinchoninic acid Assay; Thermo Fisher, Waltham MA, USA). Equal amounts of protein (30 µg) were then distributed in ready‐to‐use 4%–20% polyacrylamide gels (Bio‐Rad) and separated by electrophoresis. Proteins were then electrophoretically transferred from the gels to nitrocellulose membranes using a Trans‐Blot Turbo System (Bio‐Rad) and blocked for 1 hr with 5 g/L nonfat dry milk in PBS, containing 0.5 ml/L Tween^®^ 20 (Sigma‐Aldrich). Membranes were incubated overnight at 4°C with antibodies diluted in the same blocking buffer (1/100 for APP/Aβ, 1/500 for caspase 3, 1/1,000 for phospho‐bcl2, and 1/1,000 for BAX), followed by a 1‐hr incubation with horseradish peroxidase (HRP)‐coupled secondary anti‐mouse or anti‐rabbit antibodies (Abcam). Bound antibodies were visualized using the ECL Prime Western Blotting Detection System (Sigma‐Aldrich, Germany) and imaged with the Azure C400 Imaging System (Azure Biosystems, Dublin, CA, USA).

### 4.7 Miscellaneous protein measurements

ELISA for Aβ and BACE1 was conducted using commercial kits and according to the manufacturer's guidelines (Human Amyloid Beta 1–42 KHB3442, Thermo Fisher, Waltham Ma., USA, and Euromedex France Mouse BACE1 Kit SEA718MU).

### 4.8 Multiplex analyses for cytokines

Luminex assays were conducted on protein extracts from cell cultures and mouse tissue, using commercial kits (Bio‐Plex Pro, Mouse Chemokines Assay, Bio‐Rad; Hercules, CA, USA) following the supplier's instructions, and then, the 96‐well plates were read using a MAGPIX detection system (Luminex).

### 4.9 Immunohistochemistry

Aβ immunogenicity was evaluated using mouse monoclonal MOAB antibody (Abcam) and microglia activation with rabbit polyclonal IBA1 antibody (Thermo Fisher). Tissue was fixed in formalin and embedded in paraffin for cross‐sectioning (AML Laboratories, USA). Antigen retrieval was done at 90°C for 40 min in 0.01 M sodium citrate buffer (pH 6.0). Intrinsic peroxidases on sections were silenced with an incubation of 1 hr in 30 ml/L H_2_O_2_ (Sigma‐Aldrich, Germany). 4‐µm sections, prepared with a microtome, were then washed in PBS for 30 min followed by a 1‐hr blocking of nonspecific antigenic sites with a blocking solution consisting of PBS with 50 g/L BSA and 5 ml/L Tween^®^ 20 (Sigma‐Aldrich). Primary antibody incubation was performed in the same blocking buffer at 4°C overnight. Secondary antibody (1:500 HRP‐coupled anti‐rabbit or anti‐mouse antibodies, Thermo Fisher) incubation was performed in serum blocking buffer for 1 hr at room temperature. Slides were treated with diaminobenzidine for 3 min and mounted with Eukitt mounting medium.

### 4.10 Fluoro‐Jade C

Tissue was fixed in formalin, cryoprotected using a solution of 200 g/L glucose in PBS, and frozen at −20°C for cross‐sectioning at 20 µm. Prior to staining, sections were mounted onto slides and air‐dried for 20 min. Slides were first immersed in a basic alcohol solution consisting of 10 g/L sodium hydroxide in 800 ml/L ethanol for 5 min, followed by two rinses of 2 min in 700 ml/L ethanol and 2 min in distilled water. Slides were then incubated in 60 mg/L potassium permanganate solution for 15 min. Following a 2‐min water rinse, slides were transferred for 15 min to a 1 mg/L solution of Fluoro‐Jade C (Sigma‐Aldrich, Germany) in 1 ml/L acetic acid vehicle. The slides were then rinsed through three 1‐min changes of distilled water. Excess water was drained onto a paper towel, and the slides were air‐dried for 10 min. Slides were then mounted with VECTASHIELD Mounting Medium with DAPI (Vector Laboratories, USA).

### 4.11 Image acquisition

Staining quantification (% area stained of total area examined) was carried out using ImageJ 1.48v software (National Institutes of Health, USA). Seven nonoverlapping images of the cortex and one image of the subiculum at magnification ×40 were taken from each slide of tissue for quantification using a Zeiss microscope.

### 4.12 PET‐CT imaging

Synthesis and radiolabeling of the ^18^F‐florbetaben probe were performed as previously described (Barthel et al., 2011) and was provided by the cyclotron and radiochemistry facility at Paris Diderot University. Mice were anesthetized with 2% isoflurane in 2 L/min oxygen, injected with ^18^F‐florbetaben (10 MBq) via the tail vein, and positioned in the prone position. PET images were then acquired 90 min postinjection over 45 min in 3D list mode on a Siemens Inveon hybrid PET‐CT System (axial field‐of‐view of 12.7 cm with a bore diameter of 12 cm, approximately 1.4 mm full width at half‐maximum spatial resolution; Siemens Healthcare, Erlangen, Germany). During the scan, a heating pad prevented hypothermia. PET images were reconstructed using Pmod software (Pmod Technologies, Zurich, Switzerland), OSEM3D, 12 iterations, matrix: 256 × 256. Attenuation and scatter corrections were applied. The PET images were coregistered with the MRI template. Cortical and cerebellum volumes of interest (VOI) were generated. Global cortex to cerebellum SUVr was calculated, and means were compared between groups using a Mann–Whitney test.

### 4.13 Statistical analysis

All statistical tests used were two‐sided. The tests used were dependent on the number of groups being compared and the number of samples in each group. The data distribution was first tested for normality (Shapiro–Wilk test). If distribution was normal, parametric tests were used (Student's *t* test or ANOVA); if normality test failed, nonparametric tests were used (Mann–Whitney or Kruskal and Wallis). For three or more groups, Tukey's or Dunn's post hoc tests were used to assess differences between specific groups. Statistical analysis was completed using Prism 7.03 (GraphPad Software). All values are represented as the means ± *SEM*. No data were excluded from analysis.

## CONFLICT OF INTEREST

Dr. Tible has received a grant from Fondation Philippe Chatrier. Dr. Mouton Liger, Dr. Schmitt, Dr. Giralt, Dr. Farid, Ms Thomasseau, Dr. Gourmaud, and Pr. Paquet reported no biomedical financial interests or potential conflicts of interest. Dr. Rondi Reig has received a grant from Agence Nationale de la Recherche: MALZ 2013. Dr. Meurs has received a grant from Agence Nationale de la Recherche: MALZ 2013. Dr. Girault has received a grant from Agence Nationale de la Recherche: MALZ 2013 and Inserm. Pr. Hugon has received a grant from Agence Nationale de la Recherche: MALZ 2013 and Inserm.

## AUTHOR'S CONTRIBUTIONS

JH, JAG, EM, and LRR conceived the research, obtained the funding, assessed the results, and wrote the manuscript, MT, FML, JS, KF, ST, and CP performed the experiments. All authors reviewed and corrected the manuscript.

## References

[acel12887-bib-0001] Abraham, W. C. , & Williams, J. M. (2008). LTP maintenance and its protein synthesis‐dependence. Neurobiology of Learning and Memory, 89(3), 260–268. 10.1016/j.nlm.2007.10.001. 17997332

[acel12887-bib-0002] Barthel, H. , Gertz, H. J. , Dresel, S. , Peters, O. , Bartenstein, P. , Buerger, K. , … Sabari, O. (2011). Cerebral amyloid‐beta PET with florbetaben (18F) in patients with Alzheimer's disease and healthy controls: A multicentre phase 2 diagnostic study. The Lancet Neurology, 10(5), 424–435. 10.1016/S1474-4422(11)70077-1. 21481640

[acel12887-bib-0003] Bonnet, M. C. , Weil, R. , Dam, E. , Hovanessian, A. G. , & Meurs, E. F. (2000). PKR stimulates NF‐kappaB irrespective of its kinase function by interacting with the IkappaB kinase complex. Molecular and Cellular Biology, 20(13), 4532–4542. 10.1128/MCB.20.13.4532-4542.2000 10848580 PMC85837

[acel12887-bib-0004] Bose, A. , Mouton‐Liger, F. , Paquet, C. , Mazot, P. , Vigny, M. , Gray, F. , & Hugon, J. (2011). Modulation of tau phosphorylation by the kinase PKR: Implications in Alzheimer's disease. Brain Pathology, 21(2), 189–200. 10.1111/j.1750-3639.2010.00437.x. 21029237 PMC8094269

[acel12887-bib-0005] Carret‐Rebillat, A. S. , Pace, C. , Gourmaud, S. , Ravasi, L. , Montagne‐Stora, S. , Longueville, S. , … Hugon, J. (2015). Neuroinflammation and Abeta accumulation linked to systemic inflammation are decreased by genetic PKR down‐regulation. Scientific Reports, 5, 8489 10.1038/srep08489. 25687824 PMC4330547

[acel12887-bib-0006] Chang, R. C. , Suen, K. C. , Ma, C. H. , Elyaman, W. , Ng, H. K. , & Hugon, J. (2002). Involvement of double‐stranded RNA‐dependent protein kinase and phosphorylation of eukaryotic initiation factor‐2alpha in neuronal degeneration. Journal of Neurochemistry, 83(5), 1215–1225. 12437593 10.1046/j.1471-4159.2002.01237.x

[acel12887-bib-0007] Chang, R. C. , Wong, A. K. , Ng, H. K. , & Hugon, J. (2002). Phosphorylation of eukaryotic initiation factor‐2alpha (eIF2alpha) is associated with neuronal degeneration in Alzheimer's disease. NeuroReport, 13(18), 2429–2432. 10.1097/01.wnr.0000048020.74602.bb. 12499843

[acel12887-bib-0008] Dabo, S. , & Meurs, E. F. (2012). dsRNA‐dependent protein kinase PKR and its role in stress, signaling and HCV infection. Viruses, 4(11), 2598–2635. 10.3390/v4112598. 23202496 PMC3509664

[acel12887-bib-0009] Dumurgier, J. , Mouton‐Liger, F. , Lapalus, P. , Prevot, M. , Laplanche, J.‐L. , Hugon, J. , & Paquet, C. (2013). Cerebrospinal fluid PKR level predicts cognitive decline in Alzheimer's disease. PLoS ONE, 8(1), e53587 10.1371/journal.pone.0053587. 23320095 PMC3539966

[acel12887-bib-0010] Duyckaerts, C. , Delatour, B. , & Potier, M. C. (2009). Classification and basic pathology of Alzheimer disease. Acta Neuropathologica, 118(1), 5–36. 10.1007/s00401-009-0532-1. 19381658

[acel12887-bib-0011] Ennaceur, A. , Neave, N. , & Aggleton, J. P. (1997). Spontaneous object recognition and object location memory in rats: The effects of lesions in the cingulate cortices, the medial prefrontal cortex, the cingulum bundle and the fornix. Experimental Brain Research, 113(3), 509–519. 10.1007/PL00005603 9108217

[acel12887-bib-0012] Harding, H. P. , Zhang, Y. , Zeng, H. , Novoa, I. , Lu, P. D. , Calfon, M. , … Ron, D. (2003). An integrated stress response regulates amino acid metabolism and resistance to oxidative stress. Molecular Cell, 11(3), 619–633. 10.1016/S1097-2765(03)00105-9 12667446

[acel12887-bib-0013] Hugon, J. , Mouton‐Liger, F. , Dumurgier, J. , & Paquet, C. (2017). PKR involvement in Alzheimer's disease. Alzheimer's Research & Therapy, 9(1), 83 10.1186/s13195-017-0308-0. PMC5629792 28982375

[acel12887-bib-0014] Ill‐Raga, G. , Palomer, E. , Wozniak, M. A. , Ramos‐Fernandez, E. , Bosch‐Morato, M. , Tajes, M. , … Munoz, F. J. (2011). Activation of PKR causes amyloid ss‐peptide accumulation via de‐repression of BACE1 expression. PLoS ONE, 6(6), e21456 10.1371/journal.pone.0021456. 21738672 PMC3125189

[acel12887-bib-0015] Jawhar, S. , Trawicka, A. , Jenneckens, C. , Bayer, T. A. , & Wirths, O. (2012). Motor deficits, neuron loss, and reduced anxiety coinciding with axonal degeneration and intraneuronal Abeta aggregation in the 5XFAD mouse model of Alzheimer's disease. Neurobiology of Aging, 33(1), 196 e129–e140. 10.1016/j.neurobiolaging.2010.05.027. 20619937

[acel12887-bib-0016] Jiang, Z. , Belforte, J. E. , Lu, Y. , Yabe, Y. , Pickel, J. , Smith, C. B. , … Nakazawa, K. (2010). eIF2alpha Phosphorylation‐dependent translation in CA1 pyramidal cells impairs hippocampal memory consolidation without affecting general translation. Journal of Neuroscience, 30(7), 2582–2594. 10.1523/JNEUROSCI.3971-09.2010. 20164343 PMC2836228

[acel12887-bib-0017] Kang, R. , & Tang, D. (2012). PKR‐dependent inflammatory signals. Science Signalling, 5(247), pe47 10.1126/scisignal.2003511. PMC3656404 23092889

[acel12887-bib-0018] Marchal, J. A. , Lopez, G. J. , Peran, M. , Comino, A. , Delgado, J. R. , Garcia‐Garcia, J. A. , … Garcia, M. A. (2014). The impact of PKR activation: From neurodegeneration to cancer. The FASEB Journal, 28(5), 1965–1974. 10.1096/fj.13-248294. 24522206

[acel12887-bib-0019] Mercado, G. , & Hetz, C. (2017). Drug repurposing to target proteostasis and prevent neurodegeneration: Accelerating translational efforts. Brain, 140(6), 1544–1547. 10.1093/brain/awx107. 28549133

[acel12887-bib-0020] Mouton‐Liger, F. , Paquet, C. , Dumurgier, J. , Bouras, C. , Pradier, L. , Gray, F. , & Hugon, J. (2012). Oxidative stress increases BACE1 protein levels through activation of the PKR‐eIF2alpha pathway. Biochimica Et Biophysica Acta, 1822(6), 885–896. 10.1016/j.bbadis.2012.01.009. 22306812

[acel12887-bib-0021] Mouton‐Liger, F. , Paquet, C. , Dumurgier, J. , Lapalus, P. , Gray, F. , Laplanche, J.‐L. , & Hugon, J. (2012). Increased cerebrospinal fluid levels of double‐stranded RNA‐dependant protein kinase in Alzheimer's disease. Biological Psychiatry, 71(9), 829–835. 10.1016/j.biopsych.2011.11.031. 22281122

[acel12887-bib-0022] Mouton‐Liger, F. , Rebillat, A. S. , Gourmaud, S. , Paquet, C. , Leguen, A. , Dumurgier, J. , … Hugon, J. (2015). PKR downregulation prevents neurodegeneration and beta‐amyloid production in a thiamine‐deficient model. Cell Death & Disease, 6, e1594 10.1038/cddis.2014.552. 25590804 PMC4669750

[acel12887-bib-0023] Oakley, H. , Cole, S. L. , Logan, S. , Maus, E. , Shao, P. , Craft, J. , … Vassar, R. (2006). Intraneuronal beta‐amyloid aggregates, neurodegeneration, and neuron loss in transgenic mice with five familial Alzheimer's disease mutations: Potential factors in amyloid plaque formation. Journal of Neuroscience, 26(40), 10129–10140. 10.1523/JNEUROSCI.1202-06.2006. 17021169 PMC6674618

[acel12887-bib-0024] Page, G. , Rioux Bilan, A. , Ingrand, S. , Lafay‐Chebassier, C. , Pain, S. , Perault Pochat, M. C. , … Hugon, J. (2006). Activated double‐stranded RNA‐dependent protein kinase and neuronal death in models of Alzheimer's disease. Neuroscience, 139(4), 1343–1354. 10.1016/j.neuroscience.2006.01.047. 16581193

[acel12887-bib-0025] Paquet, C. , Mouton‐Liger, F. , Meurs, E. F. , Mazot, P. , Bouras, C. , Pradier, L. , … Hugon, J. (2012). The PKR activator PACT is induced by Abeta: Involvement in Alzheimer's disease. Brain Pathology, 22(2), 219–229. 10.1111/j.1750-3639.2011.00520.x. 21790829 PMC8029131

[acel12887-bib-0026] Patel, R. C. , & Sen, G. C. (1998). PACT, a protein activator of the interferon‐induced protein kinase, PKR. The EMBO Journal, 17(15), 4379–4390. 10.1093/emboj/17.15.4379. 9687506 PMC1170771

[acel12887-bib-0027] Rondi‐Reig, L. , Petit, G. H. , Tobin, C. , Tonegawa, S. , Mariani, J. , & Berthoz, A. (2006). Impaired sequential egocentric and allocentric memories in forebrain‐specific‐NMDA receptor knock‐out mice during a new task dissociating strategies of navigation. Journal of Neuroscience, 26(15), 4071–4081. 10.1523/JNEUROSCI.3408-05.2006. 16611824 PMC6673881

[acel12887-bib-0028] Sadleir, K. R. , Popovic, J. , & Vassar, R. (2018). ER stress is not elevated in the 5XFAD mouse model of Alzheimer's disease. Journal of Biological Chemistry, 293(48):18434‐18443. 10.1074/jbc.RA118.005769. 30315100 PMC6290164

[acel12887-bib-0029] Scheltens, P. , Blennow, K. , Breteler, M. M. , de Strooper, B. , Frisoni, G. B. , Salloway, S. , & Van der Flier, W. M. (2016). Alzheimer's disease. Lancet, 388(10043), 505–517. 10.1016/S0140-6736(15)01124-1. 26921134

[acel12887-bib-0030] Scheper, W. , & Hoozemans, J. J. (2015). The unfolded protein response in neurodegenerative diseases: A neuropathological perspective. Acta Neuropathologica, 130(3), 315–331. 10.1007/s00401-015-1462-8. 26210990 PMC4541706

[acel12887-bib-0031] Selkoe, D. J. , & Hardy, J. (2016). The amyloid hypothesis of Alzheimer's disease at 25 years. EMBO Molecular Medicine, 8(6), 595–608. 10.15252/emmm.201606210. 27025652 PMC4888851

[acel12887-bib-0032] Shimazawa, M. , Ito, Y. , Inokuchi, Y. , & Hara, H. (2007). Involvement of double‐stranded RNA‐dependent protein kinase in ER stress‐induced retinal neuron damage. Investigative Ophthalmology & Visual Science, 48(8), 3729–3736. 10.1167/iovs.06-1122. 17652745

[acel12887-bib-0033] Suen, K. C. , Yu, M. S. , So, K. F. , Chang, R. C. , & Hugon, J. (2003). Upstream signaling pathways leading to the activation of double‐stranded RNA‐dependent serine/threonine protein kinase in beta‐amyloid peptide neurotoxicity. Journal of Biological Chemistry, 278(50), 49819–49827. 10.1074/jbc.M306503200. 12975376

[acel12887-bib-0034] Tamagno, E. , Parola, M. , Bardini, P. , Piccini, A. , Borghi, R. , Guglielmotto, M. , … Tabaton, M. (2005). Beta‐site APP cleaving enzyme up‐regulation induced by 4‐hydroxynonenal is mediated by stress‐activated protein kinases pathways. Journal of Neurochemistry, 92(3), 628–636. 10.1111/j.1471-4159.2004.02895.x. 15659232

[acel12887-bib-0035] Tronel, C. , Page, G. , Bodard, S. , Chalon, S. , & Antier, D. (2014). The specific PKR inhibitor C16 prevents apoptosis and IL‐1beta production in an acute excitotoxic rat model with a neuroinflammatory component. Neurochemistry International, 64, 73–83. 10.1016/j.neuint.2013.10.012. 24211709

[acel12887-bib-0036] Yang, Y. L. , Reis, L. F. , Pavlovic, J. , Aguzzi, A. , Schafer, R. , Kumar, A. , … Weissmann, C. (1995). Deficient signaling in mice devoid of double‐stranded RNA‐dependent protein kinase. EMBO Journal, 14(24), 6095–6106. 10.1002/j.1460-2075.1995.tb00300.x 8557029 PMC394734

[acel12887-bib-0037] Zhu, P. J. , Huang, W. , Kalikulov, D. , Yoo, J. W. , Placzek, A. N. , Stoica, L. , … Costa‐Mattioli, M. (2011). Suppression of PKR promotes network excitability and enhanced cognition by interferon‐gamma‐mediated disinhibition. Cell, 147(6), 1384–1396. 10.1016/j.cell.2011.11.029. 22153080 PMC3569515

