# Supplementary material for: PKR knockout in the 5xFAD model of Alzheimer's disease reveals beneficial effects on spatial memory and brain lesions
Source: Aging Cell. 2019 Mar 1;18(3):e12887. doi: 10.1111/acel.12887 (PMC6516179; doi:10.1111/acel.12887)
Supplement: Supplementary file 1 [file ACEL-18-e12887-s001.docx]

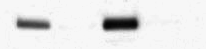

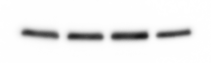


**WT**

**PKRKO**

**5xFAD**

**DM**

**PKR**

**GAPDH**

**Supplementary figure 1.** Western blot results of the brains of WT (littermates), PKRKO, 5XFAD and double mutant mice showing the absence of total PKR in PKRKO and double mutant mice.

*

**

**Supplementary figure 2.** Number of errors in the starmaze test in the four groups at the first (S1 ) and second (S2) attempt. Two-ways ANOVA test and Post hoc Newman-Keuls

S1 : PKRKO vs WT : p=0,045192 KOPKRKO vs 5xFAD : p=0,007634

S2 :no significant differences WT: wild type (littermates) DM: double mutant mice

PKRKO: PKR knockout mice


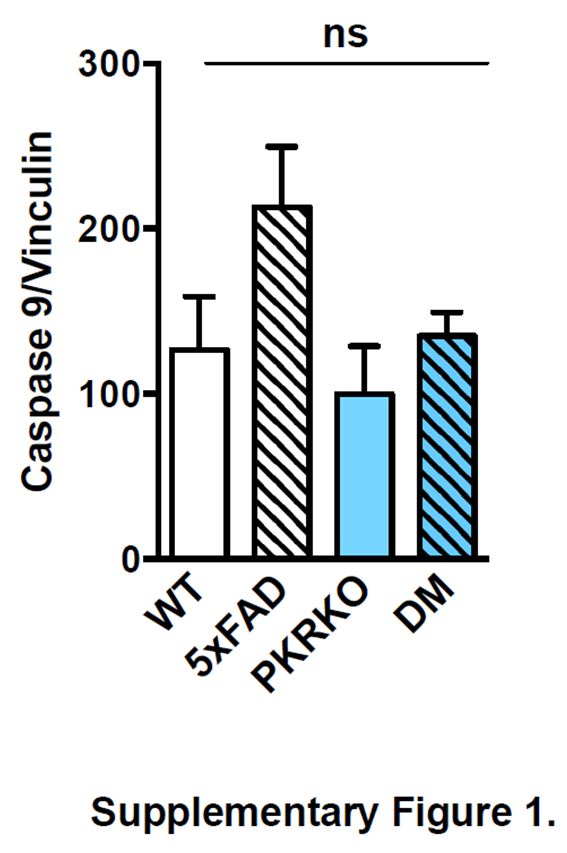


**Supplementary Figure 3**. Western blot analysis of caspase 9 in mouse hippocampus at 30 weeks; GAPDH was used as the loading control. Quantification of western blot data: mean ± SEM of n=6 mice per group. Two-ways ANOVA, p=0.076; Tukey’s post hoc test.

*p<0.05, **p<0.01, ***p<0.001, ****p<0.0001 WT: Wild Type mice DM: double mutant mice


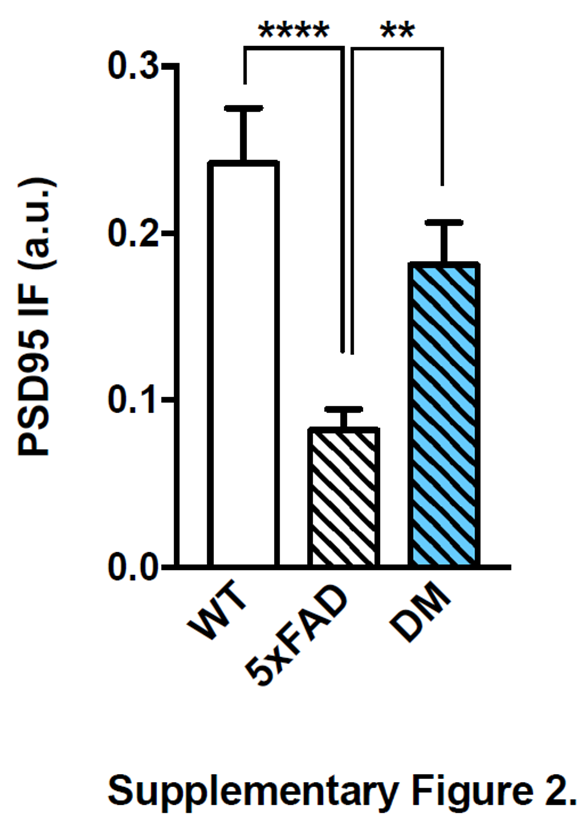


**Supplementary figure 4:** Immunofluorescence quantification of the post-synaptic protein PSD 95 immunostaining in the subiculum of mice. Mean +/- SEM of N=6 animals per group two-ways ANOVA; Tukey’s post hoc test *p<0.05, **p<0.01, ***p<0.001, ****p<0.0001 WT: Wild Type mice; DM: double mutant mice.


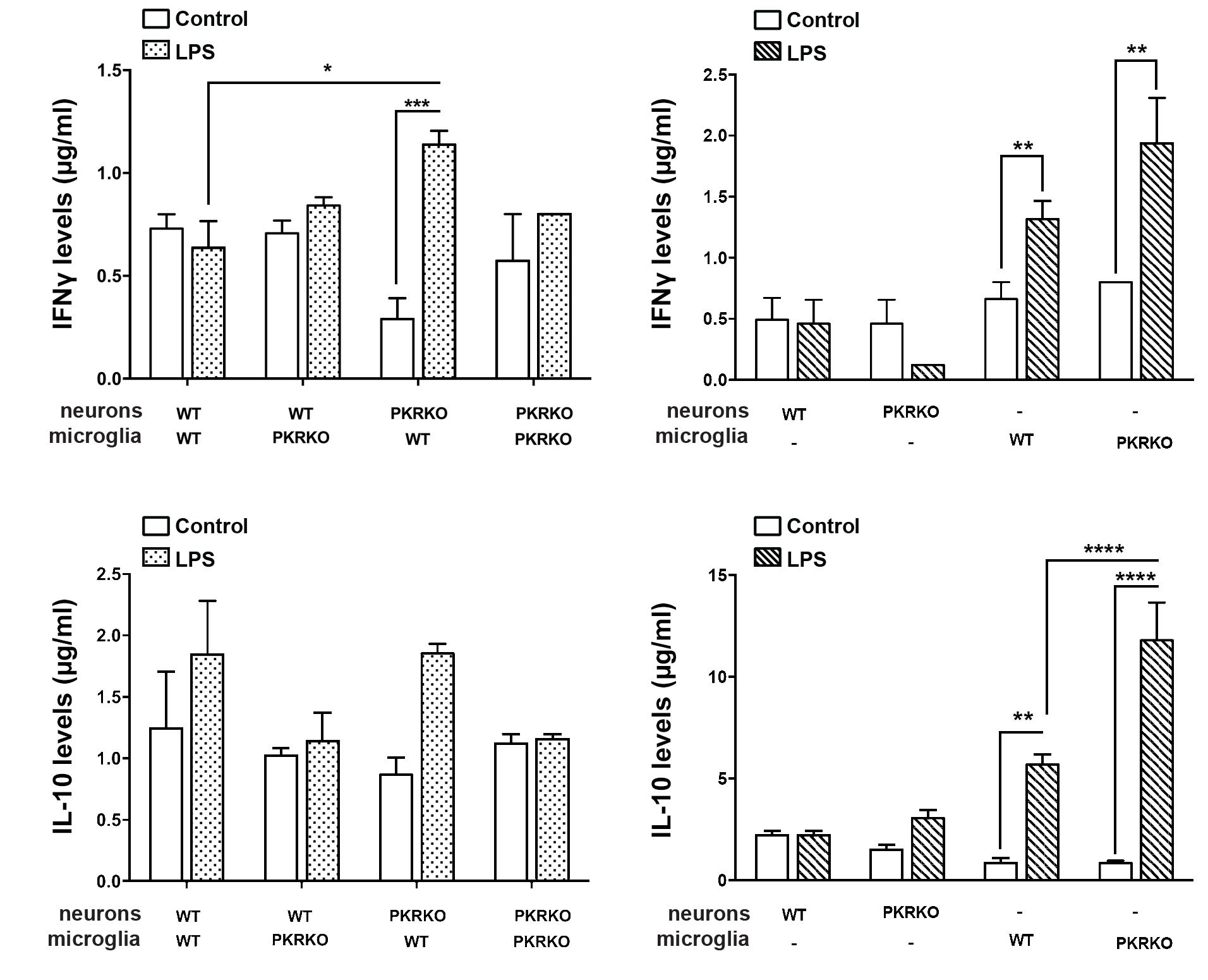


**Supplementary Figure 5 .** Luminex analysis for IFNγ and IL10 in co-cultures (left panels) and in pure primary neurons and pure microglial cultures supernatants (right panels) treated with or without LPS. Quantification of data: mean +/- SEM of n=5 supernatants per groups. Two-ways ANOVA, p=0.0030, p=0.5054 and p< 0.0001, respectively; Tukey’s post hoc test. *p<0.05, **p<0.01, ***p<0.001, ****p<0.0001


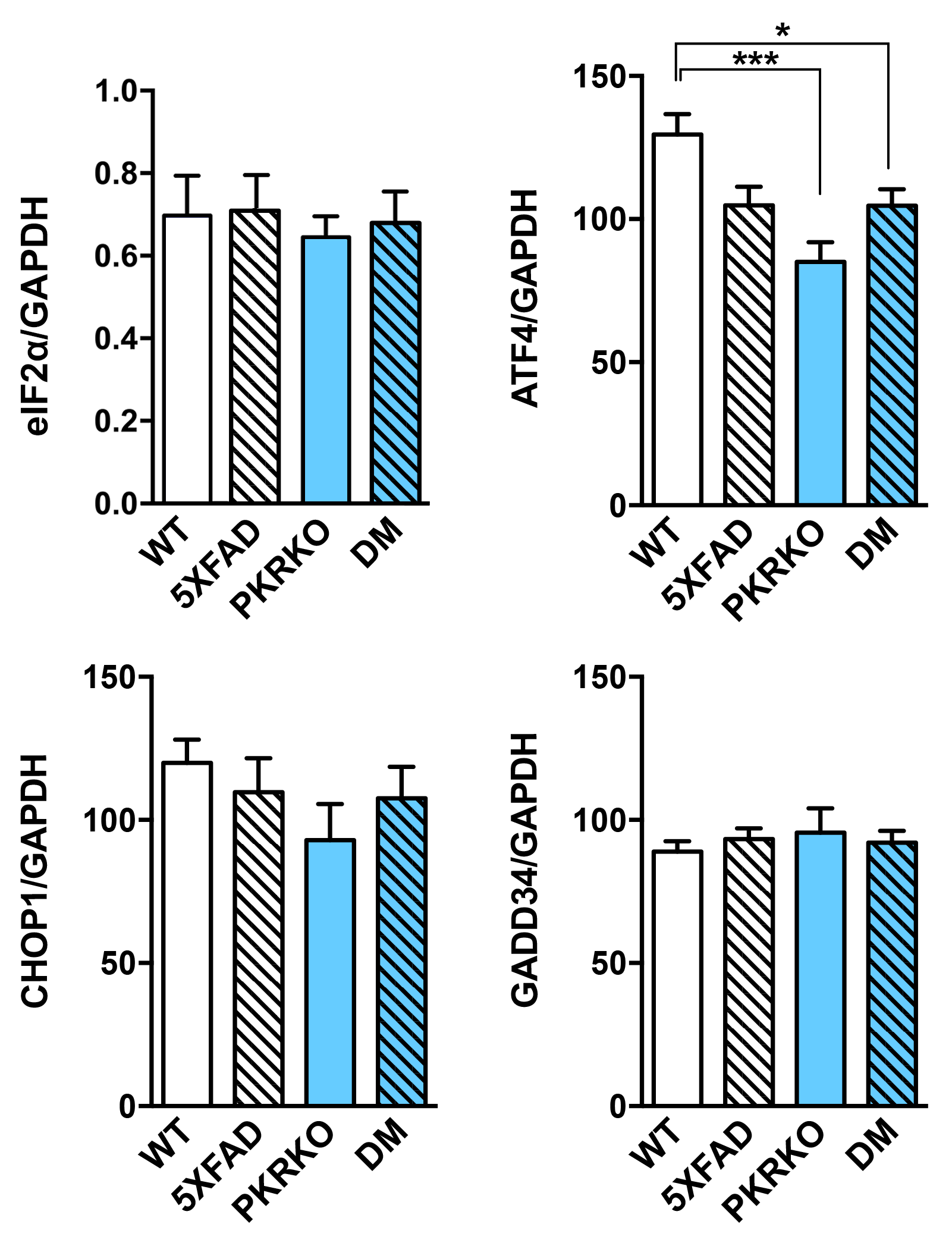


**Supplementary Figure 6**. Western blot analysis of eIF2α (a), GADD34 (b), CHOP1 (c) and ATF4 (d) in mouse hippocampus at 30 weeks, GAPDH was used as a loading control. Quantification of western blot data: mean ± SEM of n=6 mice per group. Analysis of variance (two-ways ANOVA), p=0.94, p=0.035, p=0.4088 and p=0.004, respectively; Tukey’s post hoc test. *p<0.05, ***p<0.00
